# Participant retention practices in longitudinal clinical research studies with high retention rates

**DOI:** 10.1186/s12874-017-0310-z

**Published:** 2017-02-20

**Authors:** Martha Abshire, Victor D. Dinglas, Maan Isabella A. Cajita, Michelle N. Eakin, Dale M. Needham, Cheryl Dennison Himmelfarb

**Affiliations:** 1 Johns Hopkins University School of Nursing, 525 North Wolfe Street, Suite 527, 21287 Baltimore, MD USA; 2 Outcomes After Critical Illness and Surgery (OACIS) Group, Johns Hopkins University, Baltimore, MD USA; 3 Division of Pulmonary and Critical Care Medicine, School of Medicine, Johns Hopkins University, Baltimore, MD USA; 4 Department of Physical Medicine and Rehabilitation, School of Medicine, Johns Hopkins University, Baltimore, MD USA; 5 Johns Hopkins Institute for Clinical and Translational Sciences, Johns Hopkins University, Baltimore, MD USA

**Keywords:** Retention strategies, Follow-up studies, Cohort, Longitudinal, Methods, Patient dropouts, Research design/Standards

## Abstract

**Background:**

There is a need for improving cohort retention in longitudinal studies. Our objective was to identify cohort retention strategies and implementation approaches used in studies with high retention rates.

**Methods:**

Longitudinal studies with ≥200 participants, ≥80% retention rates over ≥1 year of follow-up were queried from an Institutional Review Board database at a large research-intensive U.S. university; additional studies were identified through networking. Nineteen (86%) of 22 eligible studies agreed to participate. Through in-depth semi-structured interviews, participants provided retention strategies based on themes identified from previous literature reviews. Synthesis of data was completed by a multidisciplinary team.

**Results:**

The most commonly used retention strategies were: study reminders, study visit characteristics, emphasizing study benefits, and contact/scheduling strategies. The research teams were well-functioning, organized, and persistent. Additionally, teams tailored their strategies to their participants, often adapting and innovating their approaches.

**Conclusions:**

These studies included specialized and persistent teams and utilized tailored strategies specific to their cohort and individual participants. Studies’ written protocols and published manuscripts often did not reflect the varied strategies employed and adapted through the duration of study. Appropriate retention strategy use requires cultural sensitivity and more research is needed to identify how strategy use varies globally.

## Background

Retention of study participants is vital to ensure the power and internal validity of longitudinal research [1–3]. A high attrition rate increases the risk of bias, particularly if those lost to follow-up differ from those retained in the study or if there is differential attrition between the intervention and control groups in a randomized controlled trial (RCT) [1].

Investigation to identify effective retention strategies has increased over recent years [4]. The following retention strategy themes were identified in two systematic reviews: (1) contact and scheduling methods, (2) visit characteristics, (3) study personnel, (4) nonfinancial incentives, (5) financial incentives, (6) reminders, (7) special tracking methods, (8) study description, (9) benefits of study, (10) reimbursement, (11) study identity, and (12) community involvement [4, 5]. Studies that used multiple retention strategies had higher retention rates [4]. Despite the knowledge gained from these systematic reviews, an inherent limitation is that they were limited to published data and did not allow for in-depth exploration of retention strategies and their implementation. They potentially overlooked other retention strategies or themes that may have been effective in existing research.

The objective of this study was to identify, via survey and in-depth, semi-structured interviews, cohort retention strategies and implementation approaches used in longitudinal clinical research studies that achieved high retention rates. We intentionally focused the research question on studies that had success with high retention rates, with the goal of learning from their experience and establishing a template and tools for successful retention practices. Findings from this study augment existing information on retention strategies from previous research and systematic reviews [3–9], and inform researchers designing studies and cohort retention tools.

## Methods

A naturalistic inquiry approach was utilized to increase our understanding of retention practices in longitudinal studies that achieved high retention rates. The Johns Hopkins Medicine Institutional Review Board (IRB) database, which consists of the protocols from 7 separate IRBs with over 6500 active protocols, was queried to identify a convenience sample of studies that met the following inclusion criteria: 1) recruited ≥200 adult participants, 2) followed the participants for ≥1 year, and 3) retained ≥80% of participants using the following search: “Outcomes OR Cohort OR Longitudinal OR Long-term OR Long term OR Chronic OR Follow-up OR Follow AND Brain injury OR Chronic Obstructive Pulmonary Disease (COPD) OR Congestive Heart Failure (CHF) OR Hypertension OR “Kidney disease” OR Leukemia OR End-Stage Liver Disease (ESLD) OR Cancer OR Oncology OR Oncological OR Tumor OR End-Stage Renal Disease (ESRD) OR “Renal failure” OR Dialysis”.

Principal investigators of identified studies were contacted via an email with a description of this study and were asked to confirm study eligibility. For eligible studies, principal investigators were then asked for agreement to participate in this study and to provide contact information for a study team member who could answer detailed questions regarding the study’s retention strategies. In addition, investigators contacted members of their professional networks known to have conducted longitudinal studies within the institution. Six additional principal investigators were identified, four of whom were eligible and provided interviews. Of the 139 total studies identified through IRB query and related enquiries to established principal investigators, 22 met the inclusion criteria and 19 (86%) semi-structured interviews were completed. Reasons for not providing interviews included study completion >5 years prior, change in principal investigator, and departure of study staff responsible for retention efforts. Participants did not receive any financial incentive for participation. The Johns Hopkins Medicine Institutional Review Board approved this study with a waiver of informed consent.

### Interview and data analysis procedures

The interview guide was created, reviewed and revised by four team members with expertise in longitudinal, patient-centered outcomes research (CDH, DMN, VDD, MNE). The interview guide included collection of background information on the eligible study’s design and methodology, followed by a detailed exploration of domains of inquiry related to retention strategies. Interviews were conducted in-person or by phone by two research assistants (MA, MC), who received training on the interview guide and data collection practices prior to initiation of the study. Interview questions were provided, upon request, via an online survey before the interviews to help the interviewee gather necessary information. Two study coordinators initially responded via the online survey (Active Surveillance of Prostate Cancer, and the Longitudinal Study of Alzheimer's Disease and Other Memory Disorders), then completed the remainder of interview by phone. Prior to the interviews, the research assistants performed an online search for any study-related publications and websites that provided information on the study aims, longitudinal research visits, retention strategies and retention rates to help focus the interview. In addition, study teams were asked to provide any additional study publications or written protocols that specifically outlined retention strategies. The team of investigators reviewed the data from each interview to explore emerging common findings. According to established qualitative research methods [10], the interview guide was iteratively refined and adapted to ask subsequent study participants about additional strategies that emerged in initial interviews. Then, the data was reviewed and independently coded by four investigators (MAA, MC, MNE, CDH), using a priori themes established in prior systematic reviews (Table 1) [4, 5]. After 19 interviews, data saturation was reached. A final review of the data was performed to synthesize overarching findings from the interviews.

**Table 1:** Retention strategy themes [4, 5]

Strategies	Definition
Community Involvement	Involve community in study design, recruitment, and retention
Study Identity	Create a study identity (e.g. study logo and/or using similar colors and fonts on all study materials)
Study Personnel	Characteristics, training, and management of study personnel
Study Description	Explain study requirements and details, including potential benefits and risks to study participants
Contact and Scheduling Methods	Use of a systematic method for patient contact, appointment scheduling, and cohort retention monitoring
Reminders	Provide reminders about appointments and study participation
Visit Characteristics	Minimize participant burden through characteristics and procedures of follow-up study clinic
Benefits of Study	Provide benefits to participants and families that are directly related to the nature of the study
Financial Incentives	Provide financial incentives or payment
Reimbursements	Provide reimbursement for research-related expenses or tangible support to facilitate participation
Non-financial Incentives	Tokens of appreciation
Special Tracking Methods	Methods of tracking or dealing with hard-to-find participants

## Results

Of the 19 participating studies, 13 were prospective cohort studies, 5 were RCTs, and 1 was a quasi-experimental study (Table 2). Included studies had diverse patient populations, including victims of intimate partner violence, persons living with human immunodeficiency virus (HIV) and acquired immunodeficiency syndrome (AIDS), survivors of critical illness and other serious illnesses. The target sample size for studies ranged from 255-10,000. Actual sample recruitment at the time of interview ranged from 205 for the Community Aging in Place – Advancing Better Living for Elders (CAPABLE) study [11] with older adults aging in place to 2528 for the Alzheimer’s Disease Anti-Inflammatory Prevention Trial (ADAPT) [12]. Though each of the studies retained at least 80% of participants, 10 (53%) of the 19 studies achieved >90% retention. Five of the participating studies had published articles describing the study protocol, including some reference to retention protocols [11, 13–16]. However, none of the studies empirically tested retention strategies within their study.

**Table 2:** Characteristics of included studies

Study	PI	Study design	Study population	Target sample size	Length of follow-up (months)	Recruited sample size	Retention (%) excluding mortality unless indicated	Study duration
Longitudinal Study of the Ocular Complications of AIDS (LSOCA) [24, 25]	Thorne	Prospective Cohort	Patients with acquired immunodeficiency syndrome	2800	60	2200	93%	1998–2012
Improving Care of Acute Lung Injury Patients (ICAP) [13, 18]	Pronovost/Needham	Prospective Cohort	Patients with acute lung injury/acute respiratory distress syndrome	520	60	520	94%	2004–12
Longitudinal Study of Alzheimer’s Disease and Other Memory Disorders (LSADOMD) part of the National Institute on Aging’s Alzheimer’s Disease Research Centers [26]	Lyketsos	Prospective Cohort	People ≥60y with or without memory problems	500	Until death	500	≥80%^c^	1985-ongoing
Enroll Huntington’s Disease (Enroll-HD) [27]	Ross	Prospective Cohort	Patients with Huntington’s Disease and their family members	2285	Until death	793	≥80%^c^	2012-ongoing
Alzheimer’s Disease Anti-inflammatory Prevention Trial (ADAPT) [12]	Lyketsos	RCT	Healthy elderly individual with a family history of Alzheimer’s Disease	2625	Average 20	2528	84%^b^	2000–04
Mr. Bean - The associations between HIV, early-stage kidney disease, and cardiovascular disease [28]	Lucas	Prospective Cohort	People living with and without HIV	300	36	292	83%^b^	2010-ongoing
Active Surveillance of Prostate Cancer^a^ [16]	Carter	Prospective Cohort	Men with suspected lower grade prostate cancer	10,000	216	1369	98%	2005-ongoing
Action for Health in Diabetes Continuation (Look AHEAD-C) [29]	Clark	RCT	People living with chronic renal insufficiency	310	28	320	93%	2010-ongoing
Cohort Study to Determine Markers for the Development of Severe Vascular Events in Scleroderma^d^	Hummers	Prospective Cohort	People living with scleroderma	300	72	300	99%	2006-ongoing
Longitudinal Study of various types of Fuch’s corneal dystrophy [30]	Gottsch	Prospective Cohort	Fuch’s Corneal Dystrophy patients and family members	Not provided	Until death	Not provided	≥80%^c^	2005-ongoing
Community Aging in Place Advancing Better Living for Elders (CAPABLE) [11]	Szanton	RCT	Older adults aging in place	300	12	205	85%	2012-ongoing
Chronic Renal Insufficiency Cohort (CRIC) [20]	Appel	Prospective Cohort	People living with chronic renal insufficiency	275	24	276	99%	2002-ongoing
Study of HIV Infection in the Etiology of Lung Disease (SHIELD) [31]	Kirk	Prospective Cohort	General adult population	4000	>60	2200	≥80%^c^	2009-ongoing
Early Detection and Predicting Recurrence in Non Small Cell Lung Cancer^a^ [32]	Brock	Prospective Cohort	Non-small cell lung cancer patients	500	>60	581	≥80%^c^	2007-ongoing
Early Markers of Alzheimer’s Disease in Selected BLSA Participants: Structural and Functional Brain Changes [33]	Moghekar	Prospective Cohort	Older adults participating in the Baltimore Longitudinal Study of Aging	-	Until death	634	99%^b^	2010-ongoing
SHARE Project - Effectiveness of a Housing Intervention for Battered Women [15]	Glass	Quasi-experimental (pre/post)	Victims of intimate partner violence	300	18	278	94%	2006–2010
ARDS Network Long Term Outcome Study (ALTOS) [17, 19]	Needham	RCT	Patients with acute respiratory distress syndrome	922	12	922	94%	2008–2014
SPIROMICS - Sub-populations and intermediate outcome measures in COPD study [34]	Hansel	Prospective Cohort	Chronic obstructive pulmonary disease patients	255	36	253	81%^b^	2010-ongoing
Internet Resource for Intervention and Safety (IRIS) [21]	Glass	RCT	Victims of intimate partner violence	720	12	720	93%^b^	2011–2014

*Abbreviations*: *RCT* randomized control trial

^a^Study embedded in clinic visits

^b^Includes mortality in denominator

^c^Actual retention rate could not be provided. At least 80% retention rate per response to screening questionnaire

^d^No citation available

Table 3 provides a distribution of retention strategies employed across studies. The most commonly used strategies were from the following themes: reminders, visit characteristics, benefits of the study, and contact and scheduling. The strategy themes of study description and non-financial incentives were least reported by the participating studies. Some of the studies (Active Surveillance of Prostate Cancer study and Early Detection and Predicting Recurrence in Non Small Cell Lung Cancer study) embedded their research data collection in regular clinic visits to reduce patient burden, using the clinic protocols to serve as patient reminders. This group of clinic-based studies embedded within routine clinical care used very few strategies for recruitment and retention.

**Table 3:** Retention strategies used by each study

Strategies description [4]	Thorne, J.	Pronovost, P./Needham D.	Lyketsos, C-1	Ross, C.	Lyketsos, C-2	Lucas, G.	Carter, H.B.	Clark, J.	Hummers, L.	Gottsch, J.	Szanton, S.	Appel, L.	Kirk, G	Brock, M.	Moghekar, A.	Glass, N cohort	Needham, D.	Hansel, N.	Glass, N RCT	Total
Community Involvement					✓			✓		✓	✓	✓	✓		✓	✓			✓	10
Study Identity		✓				✓		✓		✓	✓	✓	✓			✓	✓		✓	11
Study Personnel		✓		✓	✓	✓		✓		✓	✓	✓	✓		✓	✓	✓		✓	15
Study Description		✓											✓		✓	✓	✓		✓	6
Contact and Scheduling Methods	✓	✓				✓		✓			✓	✓	✓	✓	✓	✓	✓	✓	✓	16
Reminders	✓	✓	✓	✓		✓	✓	✓	✓			✓	✓		✓	✓	✓	✓	✓	18
Visit Characteristics	✓				✓	✓		✓	✓	✓	✓	✓	✓	✓	✓	✓	✓		✓	14
Benefits of Study	✓		✓	✓		✓	✓	✓		✓		✓	✓	✓	✓	✓	✓		✓	14
Financial Incentives			✓	✓		✓		✓			✓	✓	✓			✓	✓	✓	✓	13
Reimbursements	✓	✓	✓	✓		✓		✓	✓			✓	✓			✓	✓			13
Non-financial Incentives		✓			✓			✓				✓	✓			✓			✓	7
Special Tracking Methods	✓					✓		✓	✓	✓		✓				✓	✓	✓	✓	11
Total	6	7	4	5	4	9	2	11	4	6	6	11	11	3	7	12	10	4	11	

Based on the interviews, the following were key findings associated with success in achieving high cohort retention rates: 1) cohort research staff are specialized, organized, persistent, and communicate well; 2) “personal touches” matter: tailoring retention strategies to individuals; and 3) written protocols do not reflect the many details of retention strategies employed during conduct of the study. Findings related to each of these three key messages are described below.

### Research staff are specialized, organized, persistent, and communicate well

The majority of studies (*n* = 11) used a team approach to retain study participants, employing several staff members to collect data and manage retention. Two of these studies employed a full-time staff member dedicated to implementing retention strategies and focused on optimizing participant follow up. Five of 11 studies allocated a specific number of participants to one team member who served as the study’s primary point of contact for that group of participants. This person was given strategic support at team meetings, which included adapting or developing new approaches to overcome retention barriers when standard methods of contacting a participant had been exhausted. Other features of these successful teams included selecting appropriate individuals with responsibility for participant contact and providing intensive training and support on study protocols, including retention techniques. In recruiting research staff for cohort retention activities, study teams often screened applicants for experience, communication skills, cultural-competence and specialized knowledge of the population (e.g. Spanish-speaking, domestic violence advocate). One research team provided training focused on providing staff with a common understanding of empathy and sensitivity, including mock interviews to prepare new staff prior to contacting participants on their own [13, 17–19].

In most study teams, participant data tracking was complex; tracking methods were used to inform retention efforts and facilitate communication among team members. Rigorous monitoring, often using spreadsheets or databases, enabled team evaluation of the success of retention strategies or conversely the need to adapt or innovate for the most difficult-to-reach participants. Detailed records were kept for the participant and at least one additional contact person. These records were updated at every participant contact. Study staff used phone numbers, email, texting, social media (e.g., Facebook) and internet searches to locate participants. Studies visited participants’ homes, used personal delivery of reminders and searched obituaries, court documents and incarceration records to locate participants. One highly experienced study coordinator developed a checklist of techniques used to search for participants to help ensure a complete and systematic search process was consistently undertaken.

Study teams ensured regular internal communication regarding cohort retention issues through meetings involving research assistants, study coordinators, data managers and principal investigators. During these meetings, study teams examined their latest recruitment and retention rates, discussed strategies for participants who were difficult to contact and provided each other support and ideas. One study mentioned the importance of setting daily participant contact goals for each team member; another study used friendly competition as a way to engage team members.

### “Personal touches” matter: tailoring retention strategies to individuals

To facilitate study visits, studies used tools and strategies tailored to the characteristics of individual participants or specific study requirements. For example, one study that required fasting prior to the visit provided snacks after the test was completed [20]. Some encouraged participants to bring items from home to make themselves comfortable, such as a blanket or pillow. Others provided meals or coffee. Two studies involving victims of intimate partner violence arranged for a safe meeting place and transportation as well as childcare [15, 21]. In many of the studies, parking and transportation were reimbursed. Studies also used financial compensation as a retention strategy, with amounts ranging from $10 for 1 h of survey response to $200 for a full day of data collection with invasive testing.

To help keep participants engaged throughout the study’s follow-up period, studies employed various methods. For instance, studies sent regular newsletters and cards for holidays, birthdays and even condolences. Many studies sent reminder letters about the study and upcoming visits at regular intervals. Lastly, many teams sought to develop strong staff-participant relationships by having the same staff throughout the follow-up period, which created long-standing relationships (e.g., decade-long) between study staff and participants. However, some studies, such as the CAPABLE study [11], did not require the same staff member to perform follow-up and yet were able to maintain a high retention rate throughout the study.

When appropriate, some studies sought to engage participants in groups, instead of individually, fostering a sense of community. For example, some studies hosted educational discussion forums where physicians or nurses led small group discussions related to disease management. Participants with Fuch’s corneal dystrophy were encouraged to invite the study team to family gatherings and reunions to provide education and testing for this genetic disorder. Other studies engaged participants through annual events, often held at entertainment venues (e.g., a dinner theater, horse racing) [20], using these events to encourage camaraderie among participants and study staff. Finally, one study invited participants to send in artwork created by the participant or their grandchildren to be used in future study correspondence and a calendar that featured different artwork for each month that was sent to each participant [20].

### Protocols do not reflect the many details of retention strategies employed

Only 3 (16%) of 19 studies provided written cohort retention protocols. Recognizing this pattern after the initial interviews, we added interview questions to explore the lack of written protocols to delineate retention strategies. Interviews revealed that existing study protocols were not detailed regarding techniques to locate and retain participants or were out of date. Since strategies were frequently adapted to reflect new or revised retention strategies tailored to individual participant’s circumstances, with many of the changes verbally discussed during team meetings, revised retention strategies were noted but not updated in the actual protocol. Notably, principal investigators (PIs) and study coordinators agreed that IRB review and approval is an important process and that they developed study protocols recognizing IRB and ethical issues. In some studies, retention protocols submitted to the IRB included general principles for follow-up, rather than a detailed protocol, due to concern that a detailed protocol would require frequent IRB submissions with amendments and not be practicable to implement. Moreover, there were conflicting perceptions regarding whether or not the IRB would be supportive of cohort retention approaches that include internet or social media searches and persistence when trying to locate hard-to-find participants.

## Discussion

The 19 studies with high cohort retention evaluated via in-depth interviews were heterogeneous in terms of design, sample size, target population and length of follow up. This heterogeneity allowed for exploration of a variety of approaches to cohort retention strategies and confirmed the 12 categories of retention strategies previously established. However, this study does provide further insight through the thematic analysis of in-depth interviews. These studies had research staff members who are specialized, persistent and collaborate well to accomplish their retention goals teams. They utilized personalized approaches and tailored retention strategies specific to participants in the study cohorts. This tailoring of strategies that are not formally documented in written form is an iterative process based largely on the experience and persistence of study coordinators. Cultural acceptability and feasibility of study and retention practices was evaluated by the expertise and experience of the study staff. This tailoring was facilitated by an understanding of social, cultural and environmental norms particular to the population studied.

Previous systematic reviews identified over 900 retention strategies across 88 studies, with higher retention attained in studies using more retention strategies [4, 5]. The present study suggests the importance of having a well-functioning research team that is organized, persistent, adaptable, and innovative. Prior studies focused on financial incentives to recruit and retain participants, finding a positive correlation between magnitude of incentive and retention rates [4, 5]. In our study, the use of financial incentives varied, yet all studies had high rates of retention, suggesting that participants also respond to additional strategies, such as facilitating visits and emphasizing other potential benefits of participating in the study. The in-depth interviews from this study confirm prior empirical analyses that demonstrated that achieving high retention rates requires use of multiple retention strategies and often many contact attempts by highly skilled staff [4, 22]. However, it is not clear which strategies are the most effective for study teams with limited budgets to implement. Though budget allocation for retention-specific activities was not assessed, most of these high retention studies were federally funded and adequately resourced; 15 of 19 studies had personnel that were trained and specialized, contributing to the high retention. Future studies should conduct comparative effectiveness evaluations regarding different budget allocations for retention to inform financial budget planning and prioritization among potential retention strategies.

While most research teams indicated that they have a retention protocol, after in-depth interviews, it became evident that the protocols were generally non-specific, often not reflecting all detailed aspects of retention practices. Moreover, the most successful study teams iteratively refined their retention processes in practice without updating protocols, and individualized them to specific participants. Maintaining updated retention protocols may be valuable for staff training and for maintaining high retention rates throughout the study, especially in the event of staff turnover. In addition, social media is in constant evolution and investigators should consider using technologies available to provide high quality longitudinal data. A recent article examined feasibility of using websites and social media for recruitment and demonstrated that a young female urban population is more likely to respond to these methods than traditional methods such as flyers and health fairs [23]. These recruitment strategies also use a small amount of study team resources, however it is unclear if this would be true for the purposes of retention.

Certain retention strategies are likely to have culturally-specific considerations. The question of appropriate methods and frequency for contact attempts is an important area for further exploration and may have variation in acceptability depending on the population. Likewise, the ethics of financial incentives have been widely discussed and remain an important ethical consideration. In addition, ethical regulation and oversight of studies has great variation within large academic centers, between institutions, and globally. In addition, IRB policies continue to evolve, particularly in response to new strategies such as digital and social media-based approaches for participant contact. Research practices and policies must ensure cultural-sensitivity and the protection of subjects while facilitating efficient and effective longitudinal research.

Findings from this study should be considered in light of several strengths and potential limitations. This study’s strength is the novelty of using in-depth interviews to obtain unpublished data from research teams to explore successful retention strategies for following large cohorts for at least one year. Moreover, a heterogeneous mix of high retention studies were included, with varying cohort sizes (205–2528 participants) and types of study population, including a national cohort study (ARDS Network Long Term Outcome Study – ALTOS) that enrolled over 900 acute respiratory distress syndrome (ARDS) survivors from more than 40 sites [17, 19]. However, this study also has potential limitations. First, only studies with ≥200 participants and retention rates ≥80% were considered; thereby, potentially limiting the comprehensiveness and generalizability of the data collected. However, all retention strategies from a prior systematic review [4] were exemplified through the interviews, offering some replication of findings. Future studies may examine how studies with low retention have used retention strategies and if practical resources can support study teams to improve retention rates. Also, in this study a convenience sample of studies were screened for eligibility from only one institution; hence additional retention strategies may be uncovered if conducted with a larger number of studies from other research centers. Social, cultural and regulatory body acceptance of the retention strategies described may vary by institution and internationally. As these retention strategies are used outside of the US, additional cultural tailoring of retention strategies may be required and should be studied empirically. Finally, the design of our study allowed us to identify strategies that were used together with a high degree of success. However, since these research teams applied multiple strategies we were not able to identify which individual strategies were superior. Given our insights regarding the need to tailor strategies to the context of the target population and other study factors, it is possible that each strategy identified could be implemented and yet retention goals still may not be met in a given study. To move the science of cohort retention forward, studies that compare retention strategies to identify best practices are needed.

There is need and ongoing effort to address the lack of attention and resources focused on cohort retention in longitudinal studies, including increasing number of studies reporting on retention strategies [4, 5]. To ensure validity and generalizability of results, future studies must consider and allocate resources for retention activities. This includes judiciously composing an effective research team specialized in implementing retention strategies. This project, undertaken as part of a National Heart, Lung, and Blood Institute-funded grant (R24HL111895) to create a national research infrastructure, aims to assist clinical researchers in achieving high retention rates by providing practical resources (see Tables 3, 4 and http://www.improvelto.com/).

**Table 4:** Overview of cohort retention toolkit^a^

Type of retention tool	Detailed explanation
Communication templates and manuals	Example telephone scripts with accompanying operation manuals and templates for written communication (e.g. reminder letters, occasion cards, newsletters). Examples of tools include: • Letter templates include: thank you, visit reminder • Card templates: thank you, get well, sympathy • Guideline for different scenarios a staff may encounter during phone contact
Retention strategies from systematic review	Searchable database of 618 retention strategies that were abstracted from an updated systematic review [4] on cohort retention methodology. These strategies are categorized into 12 themes. Themes include “special tracking methods,” “non-financial incentives,” and “visit characteristics.”
Participant contact information form	A template for collecting detailed contact information from participants and others who can be contacted if participant cannot be reached. Details include preferred name(s), email address.
Locating participants	Includes a template to log each contact attempt made to participants or their proxies as well as a checklist of search tools for difficult to find research participants. Examples of tools include: • Ideas and tools on how to locate participants • Template for detailed documentation of any contact attempts, which is useful for internal communication among research staff but also to track what worked and did not work in the last follow-up visit
Follow-up protocols	Examples of protocols to facilitate in-person and/or phone visits, and procedures outlining escalating efforts to reach participants. These tools synthesize and provide template for cohesively implementing retention activities. Examples of tools include: • Tools and techniques to facilitate an in-person visit • Methods and tips to overcome delayed visits or frequent cancellations
Staff training	Includes a template of documenting training and quality assurance steps in administering surveys. For example, factors to consider including pacing when interviewer-administered or how the interviewer responds if responder has question or is confused with a question.

^a^Further details of cohort retention tools made freely available via a National Heart, Lung and Blood Institute-funded grant (R24HL111895) are accessible at this website: http://www.improvelto.com/cohort-retention-tools/

## Conclusions

In-depth interviews with research staff from longitudinal studies with high cohort retention are instructive in complimenting existing empirical literature in understanding successful strategies for cohort retention. Our findings emphasize the importance of creating and sustaining well-functioning research teams focused on cohort retention, applying personalized approaches and tailored retention strategies, and continually evaluating, refining and documenting retention strategies through the duration of the study.

## Abbreviations

AIDS: Acquired immunodeficiency syndrome
ARDS: Acute respiratory distress syndrome
HIV: Human immunodeficiency virus
IRB: Institutional Review Board
RCT: Randomized controlled trial

## References

[1] Fewtrell MS, Kennedy K, Singhal A, Martin RM, Ness A, Hadders-Algra M (2008). How much loss to follow-up is acceptable in long-term randomised trials and prospective studies?. Arch Dis Child.

[2] Siddiqi AE, Sikorskii A, Given CW, Given B (2008). Early participant attrition from clinical trials: role of trial design and logistics. Clin Trials.

[3] Gupta A, Calfas KJ, Marshall SJ, Robinson TN, Rock CL, Huang JS (2015). Clinical trial management of participant recruitment, enrollment, engagement, and retention in the SMART study using a Marketing and Information Technology (MARKIT) model. Contemp Clin Trials.

[4] Robinson KA, Dinglas VD, Sukrithan V, Yalamanchili R, Mendez-Tellez PA, Dennison-Himmelfarb CR (2015). Updated systematic review identifies substantial number of retention strategies: using more strategies retains more study participants. J Clin Epidemiol.

[5] Robinson KA, Dennison CR, Wayman DM, Pronovost PJ, Needham DM (2007). Systematic review identifies number of strategies important for retaining study participants. J Clin Epidemiol.

[6] Hindmarch P, Hawkins A, McColl E, Hayes M, Majsak-Newman G, Ablewhite J (2015). Recruitment and retention strategies and the examination of attrition bias in a randomised controlled trial in children’s centres serving families in disadvantaged areas of England. Trials.

[7] Taylor RM, Mohain J, Gibson F, Solanki A, Whelan J, Fern LA (2015). Novel participatory methods of involving patients in research: naming and branding a longitudinal cohort study, BRIGHTLIGHT. BMC Med Res Methodol.

[8] Brueton VC, Tierney JF, Stenning S, Meredith S, Harding S, Nazareth I (2014). Strategies to improve retention in randomised trials: a Cochrane systematic review and meta-analysis. BMJ Open.

[9] Booker CL, Harding S, Benzeval M (2011). A systematic review of the effect of retention methods in population-based cohort studies. BMC Public Health.

[10] Flick U (2009). An introduction to qualitative research.

[11] Szanton SL, Wolff JW, Leff B, Thorpe RJ, Tanner EK, Boyd C (2014). CAPABLE trial: a randomized controlled trial of nurse, occupational therapist and handyman to reduce disability among older adults: rationale and design. Contemp Clin Trials.

[12] Meinert CL, McCaffrey LD, Breitner JC (2009). Alzheimer’s disease anti-inflammatory prevention trial: design, methods, and baseline results. Alzheimers Dement.

[13] Needham DM, Dennison CR, Dowdy DW, Mendez-Tellez PA, Ciesla N, Desai SV (2005). Study protocol: the Improving Care of Acute Lung Injury Patients (ICAP) study. Crit Care.

[14] Sabbagh MN, Thompson N, Tweedy D, Stipho-Majeed S, Kawas C, Connor DJ (2003). Recruitment and retention strategies for clinical trials in Alzheimer’s disease. Pharm Dev Regul.

[15] Clough A, Wagman J, Rollins C, Barnes J, Connor-Smith J, Holditch-Niolon P (2011). The SHARE project: maximizing participant retention in a logitudinal study with victims of intimate partner violence. Field Methods.

[16] Tosoian JJ, Trock BJ, Landis P, Feng Z, Epstein JI, Partin AW (2011). Active surveillance program for prostate cancer: an update of the Johns Hopkins experience. J Clin Oncol.

[17] Dinglas VD, Hopkins RO, Wozniak AW, Hough CL, Morris PE, Jackson JC (2016). One-year outcomes of rosuvastatin versus placebo in sepsis-associated acute respiratory distress syndrome: prospective follow-up of SAILS randomised trial. Thorax.

[18] Ruhl AP, Huang M, Colantuoni E, Lord RK, Dinglas VD, Chong A (2017). Health care resource use and costs in long-term survivors of ARDS: a 5-year longitudinal cohort study. Crit Care Med.

[19] Needham DM, Dinglas VD, Bienvenu OJ, Colantuoni E, Wozniak A, Rice TW (2013). One year outcomes of initial trophic vs full enteral feeding in acute lung injury patients: prospective follow-up ofthe EDEN randomized trial. BMJ.

[20] Denker M, Boyle S, Anderson AH, Appel LJ, Chen J, Fink JC (2015). Chronic Renal Insufficiency Cohort Study (CRIC): overview and summary of selected findings. Clin J Am Soc Nephrol.

[21] Eden KB, Perrin NA, Hanson GC, Messing JT, Bloom TL, Campbell JC (2015). Use of online safety decision aid by abused women: effect on decisional conflict in a randomized controlled trial. Am J Prev Med.

[22] Dinglas VD, Huang M, Sepulveda KA, Pinedo M, Hopkins RO, Colantuoni E (2015). Personalized contact strategies and predictors of time to survey completion: analysis of two sequential randomized trials. BMC Med Res Methodol.

[23] Staffileno BA, Zschunke J, Weber M, Gross LE, Fogg L, Tangney CC. The feasibility of using Facebook, craigslist, and other online strategies to recruit young African American Women for a web-based healthy lifestyle behavior change intervention. J Cardiovasc Nurs. 2016. 10.1097/JCN.0000000000000360 27428356

[24] Jabs DA, Van Natta ML, Holbrook JT, Kempen JH, Meinert CL, Davis MD (2007). Longitudinal study of the ocular complications of AIDS: 1. Ocular diagnoses at enrollment. Ophthalmology.

[25] Jabs DA, Van Natta ML, Holbrook JT, Kempen JH, Meinert CL, Davis MD (2007). Longitudinal study of the ocular complications of AIDS: 2. Ocular examination results at enrollment. Ophthalmology.

[26] Morris JC, Weintraub S, Chui HC, Cummings J, DeCarli C, Ferris S (2006). The Uniform Data Set (UDS): clinical and cognitive variables and descriptive data from Alzheimer Disease Centers. Alzheimer Dis Assoc Disord.

[27] Enroll-HD: a worldwide observational study for Huntington’s disease families. 2016. Available from: https://www.enroll-hd.org/ Accessed 8-3-2016.

[28] Bhasin B, Lau B, Atta MG, Fine DM, Estrella MM, Schwartz GJ (2013). HIV viremia and T-cell activation differentially affect the performance of glomerular filtration rate equations based on creatinine and cystatin C. PLoS One.

[29] Ryan DH, Espeland MA, Foster GD, Haffner SM, Hubbard VS, Johnson KC (2003). Look AHEAD (Action for Health in Diabetes): design and methods for a clinical trial of weight loss for the prevention of cardiovascular disease in type 2 diabetes. Control Clin Trials.

[30] Vasanth S, Eghrari AO, Gapsis BC, Wang J, Haller NF, Stark WJ (2015). Expansion of CTG18.1 Trinucleotide repeat in TCF4 is a potent driver of Fuchs’ corneal dystrophy. Invest Ophthalmol Vis Sci.

[31] Drummond MB, Merlo CA, Astemborski J, Kalmin MM, Kisalu A, Mcdyer JF (2013). The effect of HIV infection on longitudinal lung function decline among IDUs: a prospective cohort. AIDS.

[32] Brock MV, Hooker CM, Ota-Machida E, Han Y, Guo M, Ames S (2008). DNA methylation markers and early recurrence in stage I lung cancer. N Engl J Med.

[33] Chuang YF, An Y, Bilgel M, Wong DF, Troncoso JC, O’Brien RJ (2016). Midlife adiposity predicts earlier onset of Alzheimer’s dementia, neuropathology and presymptomatic cerebral amyloid accumulation. Mol Psychiatry.

[34] O’Neal WK, Anderson W, Basta PV, Carretta EE, Doerschuk CM, Barr RG (2014). Comparison of serum, EDTA plasma and P100 plasma for luminex-based biomarker multiplex assays in patients with chronic obstructive pulmonary disease in the SPIROMICS study. J Transl Med.

